# Fertility-Sparing Surgery for Ovarian Mucinous Tumors and Risk of Recurrence

**DOI:** 10.7759/cureus.107476

**Published:** 2026-04-21

**Authors:** Maria Aslam, Arfa Saqib, Yashfeen Ahmed, Anam Riaz, Aamir Syed

**Affiliations:** 1 Surgical Oncology, Shaukat Khanum Memorial Cancer Hospital and Research Centre, Lahore, PAK

**Keywords:** borderline ovarian tumor, disease-free survival, fertility-sparing surgery, mucinous ovarian tumor, ovarian carcinoma, recurrence

## Abstract

Background

Fertility-sparing surgery (FSS) is increasingly utilized in young women with early-stage ovarian tumors; however, data specific to mucinous histology and recurrence risk remain limited.

Objective

The objective of this study is to evaluate oncologic outcomes and recurrence patterns following FSS in patients with mucinous ovarian tumors.

Methods

This retrospective study included 32 patients with histologically confirmed mucinous ovarian tumors managed with FSS at Shaukat Khanum Memorial Cancer Hospital and Research Center, Lahore, Pakistan, a tertiary care center. Clinicopathological variables, surgical details, and follow-up outcomes were analyzed. Disease-free survival (DFS) and overall survival (OS) were assessed over a median follow-up of 18 months.

Results

The median age was 25-26 years (range 18-40). Histologically, 20 patients (62.5%) had mucinous borderline tumors, including 12 infiltrative subtypes, while 12 (37.5%) had mucinous carcinoma. The median tumor size in infiltrative tumors was 10 cm (range 4-30 cm). Laparotomy was performed in 68.8% of cases. At 18 months, OS was 100%, and DFS was 81.2%. Recurrence occurred in 6 patients (18.8%), predominantly in mucinous carcinoma (33.3%) and infiltrative borderline tumors (16.7%). Recurrences involved the contralateral ovary and omentoperitoneal surfaces. Tumor markers, cancer antigen 125 (CA-125) and carcinoembryonic antigen (CEA), remained normal in all patients.

Conclusion

FSS is a safe option in selected patients with mucinous ovarian tumors, demonstrating excellent survival outcomes. However, recurrence risk is influenced by histological subtype, particularly mucinous carcinoma and infiltrative borderline tumors, necessitating careful patient selection and close follow-up.

## Introduction

Epithelial ovarian cancer remains a leading cause of gynecologic cancer mortality worldwide, primarily due to delayed diagnosis and advanced-stage presentation. However, patients diagnosed with early-stage tumors in reference to the International Federation of Gynecology and Obstetrics (FIGO) have excellent outcomes, with survival rates exceeding 90% in stage I tumors [1]. Contemporary guidelines, including those from the National Comprehensive Cancer Network (NCCN) and the European Society of Gynecological Oncology (ESGO), emphasize individualized management strategies for early-stage disease, particularly for younger women seeking fertility preservation [2,3].

Approximately 10-15% of ovarian cancers occur in women of reproductive age, creating a critical clinical challenge in balancing oncological safety with preservation of reproductive potential. Fertility-sparing surgery (FSS), defined as conservation of the uterus and at least part of one ovary, has become an established option in carefully selected patients with stage I, including Ia, Ib, and Ic epithelial ovarian cancer. Recent guideline-based evidence underscores the importance of strict patient selection and long-term surveillance following FSS [2,3]. Recent ESGO/European Society of Gynecological Endoscopy (ESGE)/European Society of Human Reproduction and Embryology (ESHRE) guidelines highlight that FSS should be considered in appropriately staged patients with favorable histology and requires multidisciplinary decision-making and expert surgical staging [3,4].

Mucinous ovarian tumors represent a biologically distinct subtype of epithelial ovarian cancers, characterized by early-stage presentation, large tumor size, and frequent unilateral involvement. These features make mucinous tumors particularly suitable for conservative surgical approaches. Current evidence and international consensus guidelines support the use of FSS in stage I (Ia, Ib, Ic) mucinous ovarian carcinoma, provided that comprehensive staging excludes extra-ovarian disease to ensure a uniform cohort of early-stage, ovary-confined tumors [5,6].

Recent systematic reviews and multi-center studies (2020-2025) have demonstrated that FSS in early-stage ovarian cancer is associated with excellent oncological outcomes, with overall survival rates ranging from 88% to 100% and recurrence rates generally between 8% and 15%. Notably, mucinous histology has been identified as a subgroup with relatively higher recurrence risk, particularly in patients with stage IC disease or infiltrative growth patterns [7,8].

Despite growing acceptance, concerns persist regarding oncological safety, particularly the risk of recurrence following conservative management. Factors such as tumor stage, grade, adequacy of surgical staging, and histopathological subtype significantly influence recurrence risk [6].

Furthermore, most available data are derived from retrospective cohort studies, with limited prospective evidence focusing specifically on mucinous ovarian tumors. This has resulted in ongoing uncertainty regarding optimal selection criteria and long-term outcomes in this subgroup.

In this context, the present retrospective study aims to evaluate the oncological outcomes of FSS in patients with ovarian mucinous tumors, with particular emphasis on recurrence rates and associated risk factors.

## Materials and methods

This retrospective cohort study was conducted at Shaukat Khanum Memorial Cancer Hospital and Research Center, Lahore, Pakistan, a tertiary care oncology center. We evaluated patients diagnosed with ovarian mucinous tumors who underwent surgical management between January 2015 and December 2024.

We screened all patients with histologically confirmed primary ovarian mucinous tumors (benign, borderline, or malignant). The inclusion criteria were: women of reproductive age (≤45 years), histopathologically confirmed primary ovarian mucinous tumor (diagnosis of primary ovarian tumor was established based on clinicopathological correlation supported by immunohistochemical markers, specifically, tumors were labeled as primary ovarian in origin in the presence of typical morphological features and an immunoprofile consistent with ovarian origin, including positivity for CK7 and PAX8, and negativity for CK20, CDX2), undergone FSS, defined as the preservation of the uterus and at least part of one ovary; and a minimum follow-up duration of 18 months.

The exclusion criteria included: non-mucinous epithelial ovarian tumors, metastatic mucinous tumors (e.g., gastrointestinal origin), radical surgery at initial management, and incomplete clinical or follow-up data.

Data were extracted from electronic medical records, operative notes, and histopathology reports. Variables included demographics (age, parity), clinical characteristics, such as tumor markers cancer antigen 125 (CA-125) and carcinoembryonic antigen (CEA), surgical details (approach and completeness of staging), and histopathological features (tumor size, FIGO stage, and subtype). Follow-up data regarding recurrence status and survival outcomes were retrieved from the institutional oncology registry.

The primary outcome was the recurrence rate following FSS. Secondary outcomes included disease-free survival (DFS), overall survival (OS), and the identification of clinicopathological risk factors associated with recurrence.

Data were analyzed using Statistical Package for the Social Sciences (SPSS) (IBM Corp., Armonk, NY, USA). Categorical variables were summarized as frequencies and percentages, while continuous variables were expressed as median with range. Survival analysis was performed using the Kaplan-Meier method to estimate disease-free survival (DFS) and overall survival (OS) over an 18-month follow-up period. Survival distributions were compared across histological subgroups using the log-rank test.

Comparisons between categorical variables were performed using the chi-square test or Fisher’s exact test, as appropriate; given small expected cell frequencies, Fisher’s exact test was preferentially applied. Continuous variables between groups were assessed using the Mann-Whitney U test. 95% confidence intervals (CI) for proportions were calculated using the exact (Clopper-Pearson) method. A p-value <0.05 was considered statistically significant.

The study received formal approval from the Institutional Review Board (IRB) of Shaukat Khanum Cancer Center. All data were anonymized to maintain patient confidentiality, and the study adhered to the principles of the Declaration of Helsinki.

## Results

A total of 32 patients meeting the inclusion criteria were analyzed. The median age was 25-26 years (range: 18-40), reflecting a predominantly young reproductive-age cohort.

As presented in Table 1, mucinous borderline tumors (MBT) constituted 20/32 (62.5%) of cases, including infiltrative 12/32 (37.5%) and non-infiltrative 8/32 (25.0%) subtypes, while mucinous carcinoma accounted for 12/32 (37.5%). Tumor size was larger in infiltrative subtypes, with a median of 10 cm (range: 4-30 cm), although this difference was not statistically significant (Mann-Whitney U test, p > 0.05). 

**Table 1:** Clinicopathological characteristics of patients with mucinous tumors (n=32)

Variable	Statistics
Age (Years)	
Median (Range)	25–26 (18–40)
Histological Subtype, n (%)	
Mucinous Borderline Tumor (MBT)	20 (62.5%)
Infiltrative Type	12 (37.5%)
Non-infiltrative Type	8 (25.0%)
Mucinous Carcinoma (MC)	12 (37.5%)
Tumor Size (cm) – Infiltrative Subtype	
Median (Range)	10 (4–30)
Surgical Approach, n (%)	
Laparotomy (Open Surgery)	22 (68.8%)
Laparoscopy	10 (31.2%)
Postoperative Tumor Markers	
CA-125 & CEA	Normal (100%)

Laparotomy was performed in 22/32 (68.8%) and laparoscopy in 10/32 (31.2%), with no significant association with histology (Fisher’s exact test, p > 0.05). Postoperative CA-125 and CEA levels normalized in all patients (32; 100%), precluding comparative statistical analysis.

Recurrence outcomes are summarized in Table 2. During a minimum follow-up of 18 months, recurrence occurred in 6/32 (18.8%; 95% CI: 8.0%-34.9%). Recurrence rates differed by histological subtype, being highest in mucinous carcinoma (4/12, 33.3%) compared to infiltrative MBT (2/12, 16.7%) and absent in non-infiltrative MBT; however, there was no statistically significant association between histology and recurrence (Fisher’s exact test, p > 0.05).

**Table 2:** Recurrence patterns and survival outcomes by histology * Total number of patients included in the study cohort (n = 32)

Outcome Measure	Infiltrative MBT (n=12)	Mucinous Carcinoma (n=12)	Total (n=32*)
Recurrence Case, n	2/12	4/12	6/32
Subtype-Specific Recurrence Rate	16.70%	33.30%	18.80%
Anatomic Site of Recurrence			
Contralateral Ovary	2/2 (100% of recurrent cases)	4/4 (100% of recurrent cases)	—
Omentoperitoneal Disease	Present (2/2, 100%)	Present (4/4, 100%)	—
Overall Survival (18 Months)	12/12 (100%)	12/12 (100%)	32/32 (100%)
Disease-Free Survival (18 Months)	10/12 (83.3%)	8/12 (66.7%)	26/32 (81.2%)

**Figure 1:**
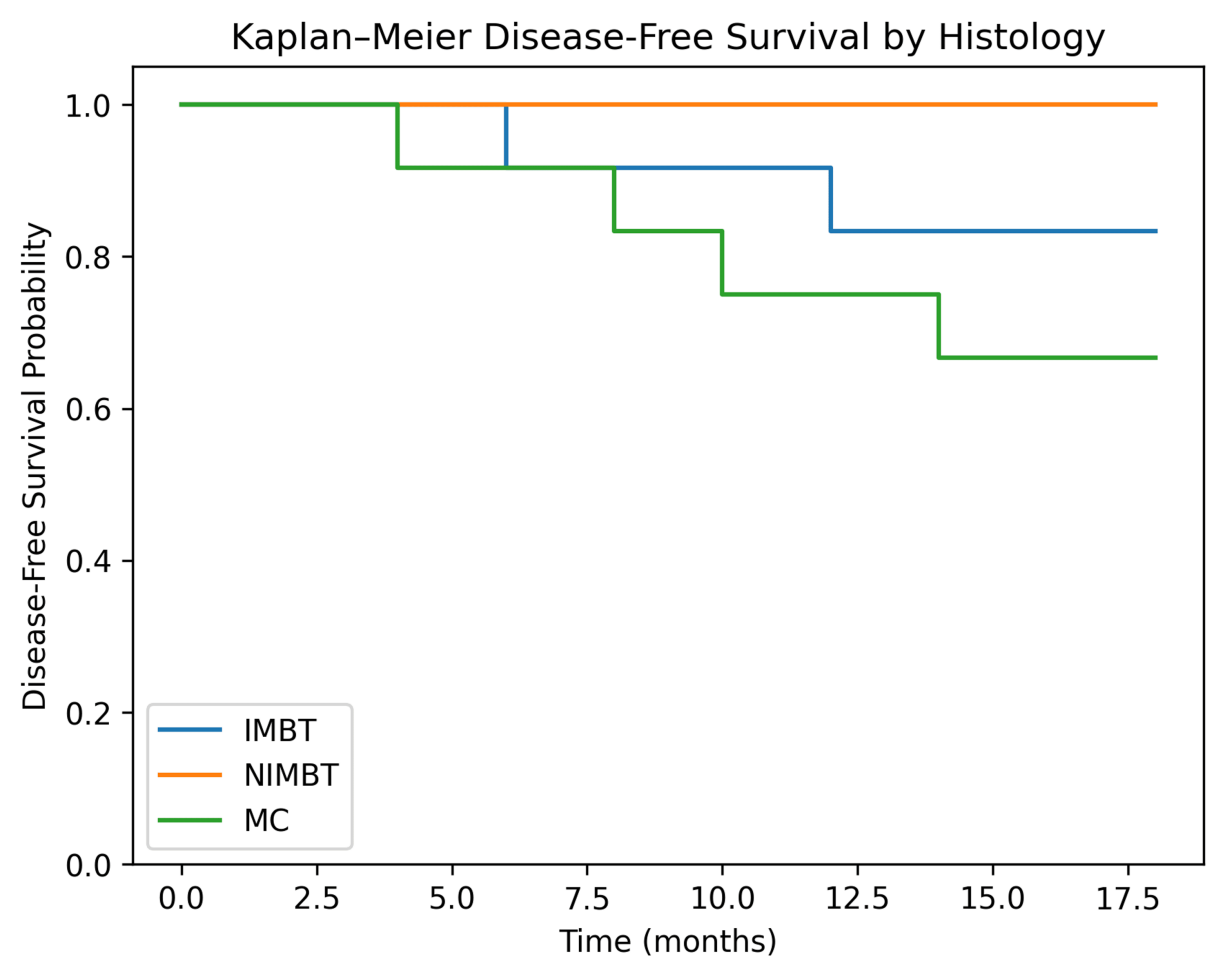
Disease-free survival by histology (Kaplan-Meier curve)

Overall survival at 18 months, as seen in Figure 1, was 32/32 (100%) across all subgroups. Disease-free survival (DFS) differed by histological subtype, being lower in mucinous carcinoma (8/12, 66.7%) as compared with infiltrative mucinous borderline tumors (10/12, 83.3%). The overall DFS was 26/32 (81.2%; 95% CI: 63.5%-92.8%), and the difference between groups was not statistically significant (p > 0.05).

As detailed in Table 2, recurrence patterns included the involvement of the contralateral ovary and omentoperitoneal surfaces. No biochemical recurrence was detected, as all patients maintained normal CA-125 and CEA levels during follow-up.

P-values were calculated using Fisher’s exact test for categorical variables due to small, expected cell counts. Continuous outcomes were not compared statistically. Survival outcomes represent proportions at 18 months and were not derived from Kaplan-Meier analysis due to limited follow-up duration and sample size.

The data specifically identifies the infiltrative MBT and mucinous carcinoma as high-risk groups, with recurrence rates of 16.7% and 33.3%, respectively, whereas non-infiltrative borderline cases showed no recurrence. The recurrence patterns, specifically omentoperitoneal disease and involvement of the contralateral ovary, suggest a risk of microscopic spread at the time of initial surgery or a biological tendency for peritoneal seeding. This confirms that for these specific aggressive subtypes, intensive monitoring is required even when postoperative tumor markers CA-125 and CEA appear normal, and there is a need for imaging in the form of a CT scan or MRI. 

## Discussion

In this retrospective study of 32 patients with mucinous ovarian tumors treated with FSS, we observed a 100% overall survival rate at 18 months and a disease-free survival rate of 81.2%, with recurrence in 18.8% of cases. These findings align with recent clinical evidence supporting the oncological safety of FSS in early-stage epithelial ovarian tumors when patients are carefully selected and comprehensively staged. Systematic reviews of FSS in epithelial ovarian cancers further affirm that FSS is associated with favorable survival outcomes, with DFS rates frequently reported above 90% across studies, though recurrence can still occur, particularly in high-risk histology [8]. The slightly lower DFS in our series may partly reflect the predominance of infiltrative and carcinoma subtypes, consistent with those risk profiles reported in larger cohorts.

A large systematic review and meta-analysis of stage I epithelial ovarian cancer demonstrated that FSS is associated with favorable oncologic outcomes and acceptable recurrence rates, comparable to radical surgery in appropriately staged patients [9]. Recent multicenter analyses of fertility-sparing approaches in borderline ovarian tumors (BOTs) show that recurrence rates after unilateral salpingoophorectomy (USO) are low, especially when complete surgical staging is performed. In a large retrospective cohort of mucinous BOTs treated at a tertiary center, there were no recurrences following USO after extended follow-up, although patients managed by ovarian cystectomy had higher residual or recurrent disease rates, emphasizing the influence of surgical technique on oncologic outcomes [10].

Contemporary literature also reflects the role of histologic subtype in recurrence risk. In early-stage mucinous carcinoma, infiltrative growth patterns have been associated with poorer outcomes compared with expansile patterns, with infiltrative tumors demonstrating comparatively higher recurrence or lower DFS rates [11]. This parallels our observation that mucinous carcinoma and infiltrative borderline tumors were over-represented among recurrences, suggesting that intrinsic tumor biology contributes significantly to relapse risk.

The normal postoperative CA-125 and CEA levels in all patients, including those with recurrence, underscore a known limitation of serum tumor markers in mucinous ovarian tumors, where they often lack sensitivity for early detection of relapse [12]. Thus, imaging and clinical surveillance remain crucial in follow-up protocols.

Overall, our study corroborates current evidence supporting FSS as a reasonable option for women with early-stage mucinous ovarian tumors who desire fertility preservation, provided that comprehensive staging is undertaken, and patients are informed about the heightened recurrence risk associated with infiltrative histology and carcinoma. Continued prospective research with larger cohorts and standardized follow-up criteria will further refine prognostic stratification and surveillance strategies in this population.

Limitations

This study is limited by its small sample size (n = 32), which reduces statistical power and limits robust subgroup analysis. Its retrospective design introduces potential selection and information bias. The heterogeneity of histological subtypes further adds biological variability that may affect outcome interpretation. The relatively short follow-up period of 18 months restricts the assessment of late recurrence and long-term survival outcomes. In addition, the low number of events limits the precision of Kaplan-Meier survival estimates. Finally, being a single-center study, the findings may not be fully generalizable to broader populations.

## Conclusions

Fertility-sparing surgery is a safe option in selected patients with mucinous ovarian tumors, showing excellent survival outcomes. However, recurrence risk remains notable and is higher in mucinous carcinoma and infiltrative borderline subtypes. Careful patient selection, adequate staging, and close follow-up are essential to optimize outcomes.
